# Interplay between Colistin Resistance, Virulence and Fitness in *Acinetobacter baumannii*

**DOI:** 10.3390/antibiotics6040028

**Published:** 2017-11-21

**Authors:** Gabriela Jorge Da Silva, Sara Domingues

**Affiliations:** 1 Faculty of Pharmacy, University of Coimbra, 3000-548 Coimbra, Portugal; saradomingues@ff.uc.pt; 2 Centre for Neurosciences and Cell Biology, University of Coimbra, 3000-548 Coimbra, Portugal

**Keywords:** *Acinetobacter baumannii*, colistin, polymyxin, antimicrobial resistance, multidrug resistance, lipopolysaccharide, lipid A, biological cost, virulence, two-component systems

## Abstract

*Acinetobacter baumannii* is an important opportunistic nosocomial pathogen often resistant to multiple antibiotics classes. Colistin, an “old” antibiotic, is now considered a last-line treatment option for extremely resistant isolates. In the meantime, resistance to colistin has been reported in clinical *A. baumannii* strains. Colistin is a cationic peptide that disrupts the outer membrane (OM) of Gram-negative bacteria. Colistin resistance is primarily due to post-translational modification or loss of the lipopolysaccharide (LPS) molecules inserted into the outer leaflet of the OM. LPS modification prevents the binding of polymyxin to the bacterial surface and may lead to alterations in bacterial virulence. Antimicrobial pressure drives the evolution of antimicrobial resistance and resistance is often associated with a reduced bacterial fitness. Therefore, the alterations in LPS may induce changes in the fitness of *A. baumannii*. However, compensatory mutations in clinical *A. baumannii* may ameliorate the cost of resistance and may play an important role in the dissemination of colistin-resistant *A. baumannii* isolates. The focus of this review is to summarize the colistin resistance mechanisms, and understand their impact on the fitness and virulence of bacteria and on the dissemination of colistin-resistant *A. baumannii* strains.

## 1. Introduction

*Acinetobacter baumannii* is an opportunistic Gram-negative pathogen recognized worldwide as a significant concern. Many isolates express diverse mechanisms of resistance that lead to the difficulty of treatment of infections caused by this microorganism. Most of the infections are in the forms of ventilator-associated pneumonia and septicaemia, especially in patients from intensive care units [1,2]. In the 1970s it was considered a low-virulence pathogen. The increased use of antibiotics and invasive methods of treatment and diagnostics in the last decades have led to the development of multidrug resistance and a rise in the frequency and severity of *A. baumannii* infections [3]. Carbapenems represent one of the last therapeutic choices for the treatment of infections due to multidrug-resistant (MDR) *A. baumannii*. Nevertheless, carbapenem-resistant isolates have been emerging [2]. Indeed, in 2017 the World Health Organization published a report where carbapenem-resistant *A. baumannii* was classified in the group of “priority 1 for research of new antibiotics” and considered as a “critical” pathogen [4]. The emergence of extensively drug-resistant (XDR) strains led to the human clinical use of an “old” antibiotic from the 1960s, colistin, which was abandoned in human therapy due to its nephrotoxicity and neurotoxicity, at a time where new antibiotics were emerging that still showed good antibacterial efficacy and less toxic effects. Since then, colistin has been mostly used in topical formulations. Nonetheless, the emergence of XDR strains has forced clinicians to use colistin as one of the last therapeutic options to fight these infections. Since the re-introduction of colistin in human clinical practice, the appearance of colistin-resistant strains has been reported [5,6,7]. Colistin resistance can be chromosomally or plasmid encoded, with the latter described recently in enterobacteria from food animals for the first time [8]. Since then, the plasmidic colistin-resistance genes *mcr*-1 to -5 have been reported worldwide in Gram-negative bacteria from human, animal, and environmental samples [7,9,10,11,12,13,14], but not in *A. baumannii*. So far, the reported mechanisms of resistance in *A. baumannii* are chromosomally encoded [15,16]. Nonetheless, the number of cases of of polymyxin-resistant *A. baumannii* strains has been increasing worldwide [16], leading to a global concern on the treatment of these infections. The majority of resistance mechanisms to colistin rely on alterations of the lipopolysaccharide (LPS), the primary target of colistin and an important virulence factor in Gram-negative bacteria. Many virulence factors have been identified in *A. baumannii* [3,17], but fundamental knowledge on virulence gene regulation and infection biology is still poorly understood [3,18].

The outcome of infections caused by drug-resistant bacteria is a complex relationship between the bacterial pathogenicity, biological cost of the resistance mutations in bacteria, host factors, and antibiotic therapy. Mutations that lead to antimicrobial resistance may modulate bacterial fitness and virulence potential. The comprehension of how antimicrobial resistance drives the biology of resistant bacterial pathogens is important to understand the outcome of an infection and the dissemination of drug resistance. Considering the clinical relevance of *A. baumannii*, some studies have been performed to evaluate the fitness cost of colistin-resistant strains.

The purpose of this review is to summarize the resistance mechanisms of *A. baumannii* to colistin and to focus on the interplay of colistin resistance, virulence, and fitness cost of the bacteria to better understand the consequences of the mutations associated with colistin resistance and the biological response of the pathogen.

## 2. Mechanism of Action of Colistin

Colistin, a bactericidal polycationic lipopeptide also known as polymyxin E, is composed of a cyclic decapeptide bound to a fatty acid chain. Its initial cellular target is the polyanionic LPS, a component of the Gram-negative bacterial outer membrane (OM). The amphiphilic feature of colistin is crucial for its interaction with the hydrophobic lipid A, a component of LPS. Lipid A has a crucial role in the control of cell permeability [19]. The electrostatic interaction between the positively-charged α,γ-diaminobutyric acid (Dab) residues of colistin and the negatively-charged phosphate groups of lipid A leads to the displacement of divalent cations Ca^2+^ and Mg^2+^, which destabilize the molecule. The alteration of the three-dimensional structure triggers the permeability of some areas of the OM, facilitating the passage of colistin through a self-promoted uptake mechanism [20]. Colistin destroys the bacterial membrane, leading to the leakage of the cytoplasmatic content and cell death [19].

Lipid A is considered an endotoxin in Gram-negative bacteria since it induces the inflammatory response with the initial release of cytokines (TNF-α) and IL-8 [21]. The anti-endotoxin effect is considered another mechanism of action of colistin by the neutralization of the LPS molecule [22].

More recently, it was reported that colistin inhibited the vital respiratory enzyme NADH-quinone oxidoreductase in the cytoplasmic membrane, although this is seen as a secondary mechanism [23].

## 3. Mechanisms of Resistance in *A. baumannii*

In *A. baumannii*, two main mechanisms of acquired resistance have been described: the modification of lipid A by adding phosphoetanolamine (PEtN) as a consequence of mutations in the *pmrA*/*pmrB* two-component system; and the complete loss of the LPS due to impaired lipid A synthesis. However, other genes that affect the biosynthesis of LPS and the structure of lipid A are also being described. Efflux pumps might also be involved in colistin resistance.

### 3.1. LPS Modification Mediated by the Two-Component System pmrA/pmrB

The most common mechanism of resistance to colistin is the modification of LPS by substitution of the phosphate groups by molecules that confer a positive charge to LPS, preventing the binding of colistin. In *A. baumannii*, the mutations in the *pmrA* and/or *pmrB* genes (more common in *pmrB*) may induce the constitutive expression of *pmrA* that leads to the up-regulation of the *pmrCAB* operon, and subsequent synthesis and addition of PEtN to the 4′-phosphate or 1′-phosphate of lipid A [24,25,26,27,28,29].

The resistant phenotype can be reverted to a susceptible one due to compensatory mutations in the *pmr locus*, decreasing the hyper-activation of the *pmrCAB* operon, or the *pmr* gene may change to its non-mutated form [26,27]. However, some strains can maintain the *pmrB* mutation, with no additional compensatory mutations. Moreover, a study reported that six out of 30 colistin-resistant *A. baumannii* strains did not evidence any mutations in the *pmrA*/*pmrB* locus. This finding suggests that other genes might be involved in the acquisition of colistin resistance, leading to the overexpression of the PmrAB two-component system. Increased expression of both genes seems to be essential for colistin resistance, in contrast with amino acid changes [30].

Other alterations of the lipid A structure were found in clinical and laboratory colistin-resistant strains that have a diphosphoryl hepta-acylated lipid A structure with both pEtN and galactosamine (GalN) modifications [31]. Hepta-acylation of lipid A promotes protection against cationic antimicrobial polipeptides, including polymyxins. In *Escherichia coli* and *Salmonella* spp. the LPS portion of the OM barrier is reinforced by the increased production of the OM acyltransferase PagP, resulting in the formation of protective hepta-acylated lipid A. *A. baumannii* does not possess the gene *pagP*, and developed a PagP-independent mechanism to synthesize the protective hepta-acylated lipid A. Two putative acyltransferases (designated LpxLAb and LpxMAb) were characterized, and transfer one and two lauroyl (C12:0) acyl chains, respectively, during lipid A biosynthesis. The LpxMAb-dependent acylation of lipid A was also shown to be essential to the survival of *A. baumannii* strains in desiccation conditions [32].

### 3.2. Loss of LPS

LPS is synthesised in the cytoplasm through the Lpx pathway and it is translocated to the OM by the Lpt pathway. Mutations by nucleotide substitution, deletion, or insertional inactivation by the insertion sequence IS*Aba11* in the genes *lpxA*, *lpbxC* and *lpxD*, which are involved in the lipid A biosynthesis, have been described in colistin-resistant *A. baumannii* strains, leading to the complete loss of LPS [33,34]. Additional mutations of the *lpsB* gene, encoding a glycosyltransferase, and involved in the synthesis of the LPS core, have also been associated to colistin resistance. The absence of lipid A, the initial target of colistin, results in high resistance to colistin [35,36]. Also, LPS may not be present in the OM due to mutations in the gene that encodes the outer membrane protein LptD, responsible for the final translocation of the LPS molecule after its synthesis [37]. The LPS-deficient bacteria alter the activation of the host innate immune inflammatory response [38].

A recent study compared the metabolome of polymyxin-susceptible and polymyxin-resistant *A. baumannii* strains, showing three quite different metabolic profiles: (1) alterations in specific amino acid and carbohydrate metabolites, particularly from the pentose phosphate pathway (PPP) and tricarboxylic acid (TCA) cycle intermediates; (2) nucleotide levels lower in the LPS-deficient strain; and (3), increased abundance of short-chain lipids compared to the parent polymyxin-susceptible ATCC 19606 (ATCC is international (American Type Culture Collection)) [39].

### 3.3. Genes Involved in the Outer-Membrane Asymmetry

Mutations in genes other than *pmrAB* and *lpxACD* may also be responsible for the change of antimicrobial susceptibility to colistin. The asymmetric distribution of lipids in the OM of Gram-negative bacteria is essential for its function as a barrier and integrity of the cell. The accumulation of phospholipids in the outer leaflet of the OM disrupts the LPS organization and increases the cell lability to small toxic molecules. The OM lipoprotein VacJ is part of the Vps-VacJ ABC transporter system responsible for maintaining the phospholipids in the OM inner leaflet and the LPS in the outer leaflet of the membrane (OM asymmetry) [40]. The activity of PldA, a phospholipase, is increased in bacteria cells with destabilized membrane and it seems to remove phospholipids from the outer leaflet of the OM to maintain asymmetry [41]. Recently, it was suggested that both *vacJ* and *pldA* genes, where mutations were identified, may play a role on the *A. baumannii* colistin resistance due to their association to the maintenance of OM asymmetry [42].

### 3.4. Efflux Pumps

A few studies suggest that efflux pumps may be involved in the colistin resistance phenotype in *A. baumannii*. Eighteen genes encoding putative efflux transporters were shown to be upregulated in response to physiological concentration of NaCl, resulting in a tolerance to diverse antibiotics, including colistin [43]. More recently, another study showed that the use of efflux inhibitors like cyanide 3-chlorophenylhydrazone (CCCP) may reduce significantly the minimal inhibitor concentrations for colistin, strongly suggesting the involvement of efflux pumps in the colistin resistance phenotype [44].

## 4. Fitness and Virulence

The biological cost conferred to the host by a resistance trait is considered a key parameter in the spread and stability of resistant bacteria. Resistance mutations typically change and/or impair targets with essential functions, and are usually associated with a fitness cost. Nonetheless, cells can often ameliorate the cost of the resistance due to compensatory mutations. The amelioration of resistance can result in the stabilization of the resistance in the bacterial population [45,46]. Fitness is usually evaluated by measurement of growth rates and/or pairwise competition experiments.

The pathogenic potential of a bacteria reflects its virulence, which is usually measured by the mortality or the host reproduction rates associated with a strain. Changes in virulence (increased or decreased) have been detected in resistant strains belonging to different species [47,48]. Some of the virulence factors that have been identified in *A. baumannii* include the outer membrane protein OmpA, phospholipases, efflux pumps, penicillin-binding proteins, and outer membrane vesicles [49,50].

Although fitness and virulence are different concepts, studies often evaluate both. Therefore, fitness and virulence will be discussed together and we will attempt to relate these traits with genetic mutations associated with colistin resistance. Some studies test clinical isolates while others expose ATCC strains to increased concentrations of colistin to generate different resistant isolates.

### 4.1. Colistin Resistance Due to Mutations in pmr Genes

Clinical *A. baumannii* isolates often acquire colistin resistance during treatment with this antibiotic [26,51,52,53]. Two clinical *A. baumannii* strains, which acquired colistin resistance after treatment with this polymyxin, were shown to have different clinical outcomes. *A. baumannii* ABIsac_ColiR [25] showed impaired virulence, as seen by loss of clinical signs of infection in the human patient [51] and in a rat model of acute pneumonia [52], while *A. baumannii* CR17 did not lose its infecting capacity [54]. Both strains were later further explored and colistin resistance was associated with *pmrA* mutations: *A. baumannii* ABIsac_ColiR with *pmrA* E8D [25] and *A. baumannii* CR17 with *pmrA* M12K [55]. Strain ABIsac_ColiR was also shown to have lost a prophage, which could contribute to or explain the loss of virulence in this strain [25]. A decreased in vitro and in vivo fitness has also been observed [52]. Although strain CR17 remained infectious, it was also associated with a decreased virulence and fitness, as compared with the initial susceptible strain CS01 [55]. The levels of virulence vary between *A. baumannii* strains [56], and the retention of the capacity to infect of CR17 strain might be related with the initial high virulence of the susceptible strain [55].

Four clinical *A. baumannii* strains showed an in vitro fitness cost, seen as the decreased growth rates of the resistant strains. The in vivo fitness cost was evidenced by the loss of resistance after treatment cessation. Although mutations in different genes were observed, all resistant isolates carried *pmrB* mutations which also varied, including P233S, R263C, M145I, T13A, or indel AAT at position 69. These mutations could be associated with the overexpression of the PmrC phosphoethanolamine transferase, with a consequent increase of the *pmr* operon transcript. Return to susceptibility to colistin occurred by different mechanisms. In two patients, there was re-emergence of the susceptible strain. In another patient, the resistant strain was lost, but unexpectedly, the apparent susceptible strain was detected to be also present during colistin treatment. This strain carried a L271R mutation in *pmrB*, associated with a low fitness cost and in vivo stability in the absence of colistin. In the fourth patient, with the resistant isolate carrying the *pmrB* P233S mutation, a compensatory mutation in *pmrA* (L206P) was observed, making the future re-acquisition of colistin resistance highly unlikely [26]. In a different study, the same *pmrB* P233S mutation present in other clinical colistin-resistant *A. baumannii* strains (Ab4451 and Ab4452) was not associated with loss of virulence or reduced fitness; compensatory mutations in the *pmrCAB* locus were also not detected. Despite the fact that in vitro and in vivo fitness costs were not observed in Ab4451 and Ab4452, resistance to colistin was lost after colistin was withdrawn [57]. In a recent study, the *pmrB* P233S mutation was not associated with a fitness cost, but had an impact on the in vitro virulence, as evaluated by attenuated proteolytic activity and siderophore production, of the clinical strain C440 [58]. While the study by Snitkin and colleagues showed mutations in other genes usually not specifically related with colistin resistance [26], Durante-Mangoni et al. only sequenced the *pmrCAB* and *lpxACD* loci [57]. Whether compensatory mutations in non-analysed genes, post-translational modifications, or physiological changes could explain the different study outcomes remains to be determined.

Two different clinical strains acquired colistin resistance after patient treatment with colistin. Ab249 and Ab347 harboured *pmrB* P233S and P170L mutations, respectively. Both strains showed a reduced in vitro fitness and in vitro and in vivo decreased virulence [53,59]. The reduced virulence could be associated with a diminished initial cell adhesion with consequent reduced ability of the resistant strains to produce biofilm. Additionally, Ab249 carried a mutation in *lpsB* (*241K), and Ab347 lost several genes while carrying a mutation in CarO (A19fs), which has been previously associated with biofilm production [59].

In contrast, decreased in vitro fitness and virulence was not observed in a clinical *A. baumannii* strain with a deletion of one amino acid in PmrB (ΔI19) [58]. In another study, where clinical strains were exposed to sub-MIC concentrations of colistin, while *pmrB* S17R mutant showed a slight decrease in fitness and virulence, these changes were not observed in strains with *pmrB* 17_26dup or T235I [60].

An in vitro-induced colistin-resistant derivative of *A. baumannii* ATCC 19606, called RC64, showed an increased in vitro [61] and in vivo fitness cost as well as impaired virulence [62]. The decreased fitness in the resistant strain has been associated with the down-regulation of several proteins, including outer membrane proteins, chaperones, protein biosynthesis factors, and metabolic enzymes [61]. The mutations R134C and A227V have been identified in the *pmrB* of this strain [62]. Low-iron conditions, such as those found in the human serum, were related with the decrease in vitro fitness [63] of strain RC64 [62] and also clinical colistin-resistant strains [64] with different *pmrB* mutations, which were not directly correlated with the reduced growth phenotype [63]. Another colistin-resistant derivative of *A. baumannii* ATCC 19606, with A227V *pmrB*, showed decreased in vitro and in vivo fitness, as well as attenuated virulence, although this was not observed in all tested models [65].

A slight decrease of the in vitro fitness of a colistin-resistant derivative of *A. baumannii* ATCC 17978 was associated with *pmrB* G272D [46].

Colistin-resistant clinical strains recovered during colistin therapy revealed a reduced in vitro fitness as determined by the growth rates and by pairwise competitions assays with their susceptible counterparts. These strains presented mutations in *pmrB* S17R, T232I, R263L, Y116H and/or *pmrA1* E8D, with the highest fitness decrease associated with co-presence of *pmrB* Y116H and *pmrA1* E8D [24,66]. Some of the strains, collected from the same patient over the course of colistin treatment (which has its bactericidal effect due to production of hydroxyl radicals) were further studied; the study included the susceptible parental strain as well as five resistant strains. Both in vitro and in vivo assays revealed that, after an initial loss of fitness and virulence, colistin-resistant isolates progressively increase their fitness as well as virulence under oxidative stress. This study also shows that in vitro results do not necessarily correlate with the in vivo outcome [66].

### 4.2. Colistin Resistance Due to Mutations in lpx Genes

Fewer studies report on the fitness and virulence of colistin-resistant *A. baumannii* due to mutations in the *lpxACD* locus. In one of the studies, in vitro and in vivo fitness costs were detected in the *lpx* mutants, with the Δ*lpxD* mutant showing the highest in vitro fitness cost, as compared with the wild-type *A. baumannii* ATCC 19606. Virulence was also evaluated in A549 human alveolar cells and in a mouse model, and in *Caenorhabditis elegans*, with the *lpx* mutants showing decreased virulence, seen as decreased mortality rate or reduced inhibition of fertility, respectively [65]. In another study, Wand and colleagues showed that single mutations in the *lpxA* (E216*), *lpxC* (I253N, F191L or A82E), or *lpxD* (K318fs) genes, or inactivation of *lpxC* (*lpxC*::IS*Aba1*), obtained after colistin exposure to clinical *A. baumannii* strains, were associated with a reduced fitness and avirulence in *Galleria mellonella* [60]. As described above, these studies also evaluated the biological costs and effect on virulence associated with mutations in the *pmrB* gene. Comparing the results, the influence of mutations in the fitness and virulence was more pronounced in the *lpx* mutants [60,65].

Mu and colleagues showed that mutation *lpxA* I76K and disruption of *lpxC* or *lpxD* by IS*Aba1* confer fitness costs to colistin-resistant derivatives of *A. baumannii* ATCC 17978, but additional mutations in other genes, such as *hepA* or *rsfS*, contributed to an increased resistance to colistin, as well as a (partially or completely) compensated fitness cost of these mutants. Additional costs were observed when mutants were grown in serum [46].

Table 1 summarizes the outcome of fitness and virulence assays in *A. baumannii* strains with mutations in known colistin-resistance genes.

## 5. Conclusions

Most of the studies report a fitness cost and a decreased virulence of colistin-resistant *A. baumannii*. However, this seems to be dependent on several factors, such as the mutated gene, the specific mutation, and the tested strain. The loss of LPS (*lpx* mutants) has a higher impact on the strain fitness and virulence in comparison with those that only have modifications of the LPS (*pmr* mutants). This is comprehensible since *lpx* mutants lack the endotoxic LPS and the cells lose the wall integrity.

Numerous mutations in the same gene are associated with colistin resistance. Diverse mutations might be associated with different outcomes, especially if they differently influence the protein structure or function [58,62]. At the same time, the same mutation might have different effects on the fitness and the virulence of the strain, which can also be associated with the existence of compensatory mutations in other genes. The plasticity of the *A. baumannii* regulation systems can also potentially affect the influence of colistin resistance on virulence [58], as well as post-translational modifications or physiological changes [66].

Another factor that can influence the fitness and virulence results is the different genetic backgrounds of the tested strains. Clinical strains are highly variable, belong to different sequence types, and carry different virulence genes, which can impact the overall virulence and fitness of each strain. Different ATCC strains also produce different outcomes [46]. To overcome this limitation, the different genes involved in colistin resistance, carrying different mutations, should be tested in the same genetic background.

The reported studies use different methods, which can also influence the result. Additionally, as recently observed, in vitro results might not correlate well with the in vivo outcome [66]. Therefore, more in vivo studies are needed.

The high fitness costs associated with colistin resistance, especially in mutants that lose LPS, may limit the spread of these isolates in clinical environment and could explain the sporadic nature of colistin-resistant *A. baumannii* outbreaks [6,64,67,68]. Nonetheless, recent evidence shows that compensatory mutations can ameliorate the biology cost triggered by colistin resistance, which can increase survival and spread in these environments. At the same time, there is some confidence in the successful treatment of colistin-resistant *A. baumannii* infections, as colistin-resistant mutants have an increased susceptibility to several antibiotics [46,69,70]. Nonetheless, this trait does not seem to be universal [24,53].

In conclusion, *A. baumannii* shows a remarkable genetic plasticity that allows a quick adaption to environmental conditions. The old paradigm that there is a trade-off between resistance and virulence might not always apply, even in colistin-resistant strains where LPS is lost or modified. The interplay between genetic virulence regulation and antimicrobial resistance is complex and seems to be highly strain-dependent. More in depth and fundamental studies are needed to fully comprehend the interaction of resistance and bacteria biology that could help in the development of interventions to control the dissemination of colistin-resistant strains.

## Figures and Tables

**Table 1:** Studies showing mutations present in well-known colistin-resistance genes and fitness cost and virulence observed in isolates.

Source of Resistance	Gene	Mutation (Amino Acid Level)	Fitness Cost		Impaired Virulence	Reference
			In Vitro	In Vivo		
Clinical	*pmrA*	E8D	Yes	Yes	Yes	[25,51,52]
Clinical	*pmrA*	M12K	Yes	Yes	Yes ^b^	[54,55]
Clinical	*pmrA1*	E8D	Yes	-	-	[24]
Clinical	*pmrB* + *pmrA1*	Y116H + E8D	Yes	-	-	[24]
Lab acquired	*pmrB*	R134C and A227V	Yes	Yes	yes	[61,62]
Lab acquired	*pmrB*	G272D	Yes ^a^	-	-	[46]
Clinical	*pmrB*	T13A; indel AAT at 69; M145I; P233S; R263C	Yes	Yes		[26]
Clinical	*pmrB*	L271R	-	Yes ^a^	-	[26]
Clinical	*pmrB*	P233S	No	No	No	[57]
Clinical	*pmrB*	P233S	No	-	Yes	[58]
Clinical	*pmrB*	P233S; P170L	Yes	-	Yes	[53,59]
Clinical	*pmrB*	ΔI19	No	-	No	[58]
Clinical	*pmrB*	S17R; T232I; R263L	Yes	-	-	[24]
Lab acquired	*pmrB*	S17R	-	Yes ^a^	Yes ^b^	[60]
Lab acquired	*pmrB*	17_26dup; T235I	-	No	No	[60]
Lab acquired	*pmrB*	A227V	Yes	Yes	Yes ^b^	[65]
Lab acquired	*lpxACD*	Δ*lpxA*; Δ*lpxC*; Δ*lpxD*	Yes	Yes	Yes	[65]
Lab acquired	*lpxA*	E216 *	-	Yes	Yes	[60]
Lab acquired	*lpxA*	I76K	Yes	-	-	[46]
Lab acquired	*lpxC*	I253N; F191L; A82E; Δ*lpxC*	-	Yes	Yes	[60]
Lab acquired	*lpxC*	Δ*lpxC*	Yes	-	-	[46]
Lab acquired	*lpxD*	K318fs	-	Yes	Yes	[60]
Lab acquired	*lpxD*	Δ*lpxD*	Yes	-	-	[46]

^a^ A slight cost was observed; ^b^ Although reduced virulence was observed, the strains did not become avirulent; * Stop codon.
